# Supplementary material for: Role of Arginase-II in Podocyte Injury under Hypoxic Conditions
Source: Biomolecules. 2022 Aug 31;12(9):1213. doi: 10.3390/biom12091213 (PMC9496188; doi:10.3390/biom12091213)
Supplement: Supplementary file 1 [file biomolecules-12-01213-s001.zip › Legend to Suppl. Video Files_Final.pdf]

Suppl. Video File Legend

A representative Z-stack recording showing co-localization of Arg-II (green) and synaptopodin (SNPT, red) in glomerulus of aged wild type mice under hypoxic conditions. White arrow indicates podocytes co-expressing both Arg-II and SNPT. Nucleoli were stained with DAPI (blue).
