# Supplementary material for: Role of Arginase-II in Podocyte Injury under Hypoxic Conditions
Source: Biomolecules. 2022 Aug 31;12(9):1213. doi: 10.3390/biom12091213 (PMC9496188; doi:10.3390/biom12091213)

Original blots for Fig. 1

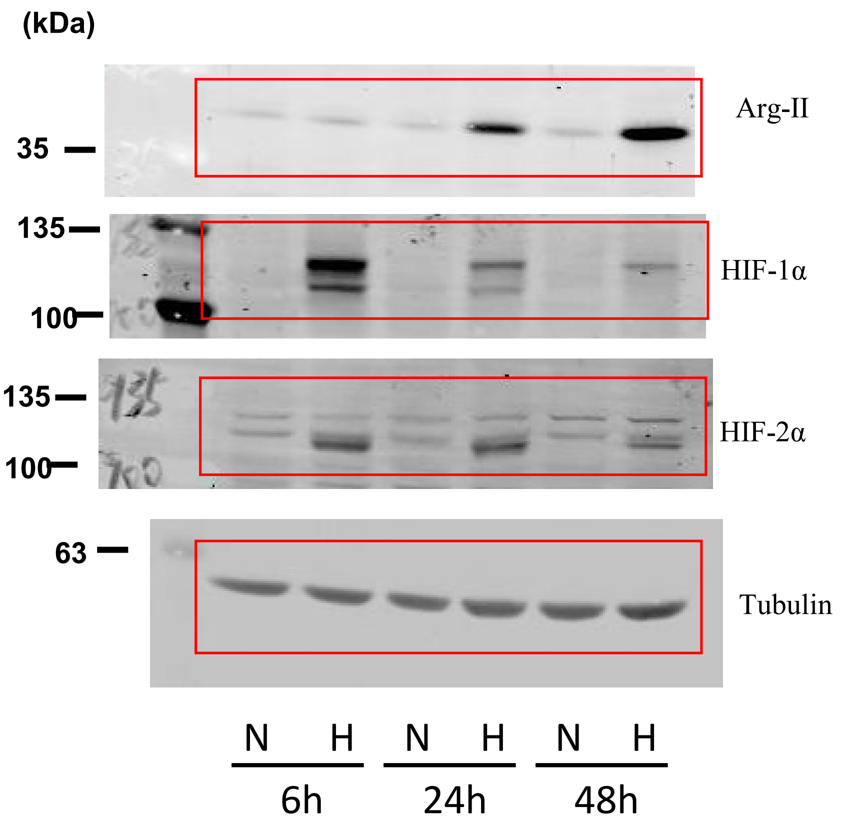

N= normoxia

H= hypoxia

Original blots for Fig. 2

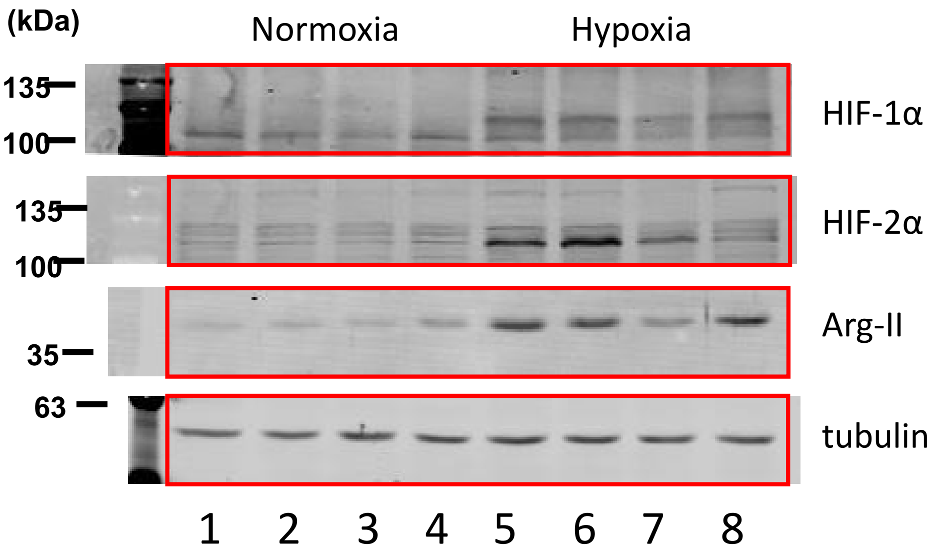

1. Normoxia without virus (-)
2. Normoxia with shRNA-LacZ
3. Normoxia with shRNA-HIF1α
4. Normoxia with shRNA-HIF2α
5. hypoxia without virus (-)
6. hypoxia with shRNA-LacZ
7. Hypoxia with shRNA-HIF1α
8. hypoxia with shRNA-HIF2α

Original blots for Fig. 3

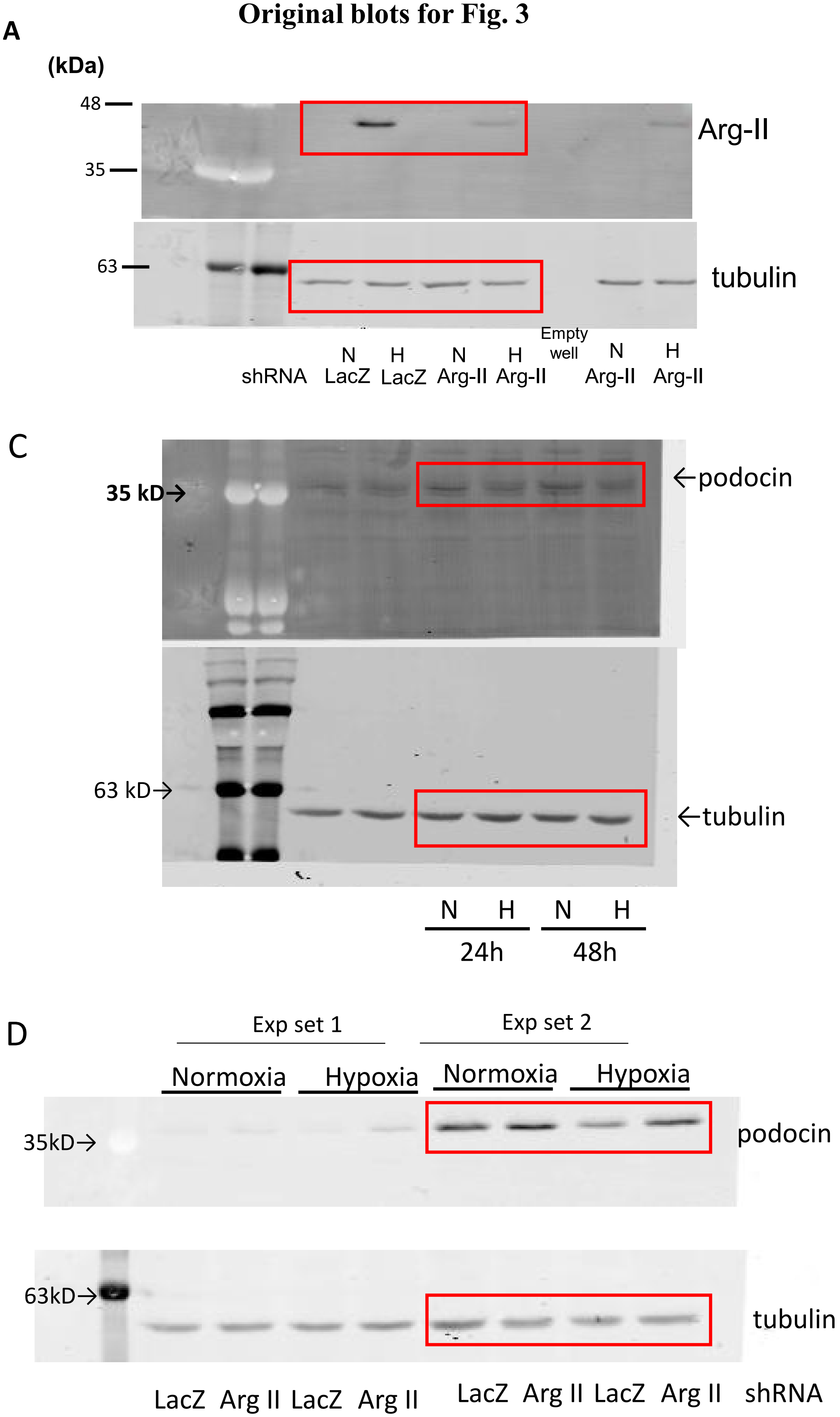

Original blots for Fig. 5

E

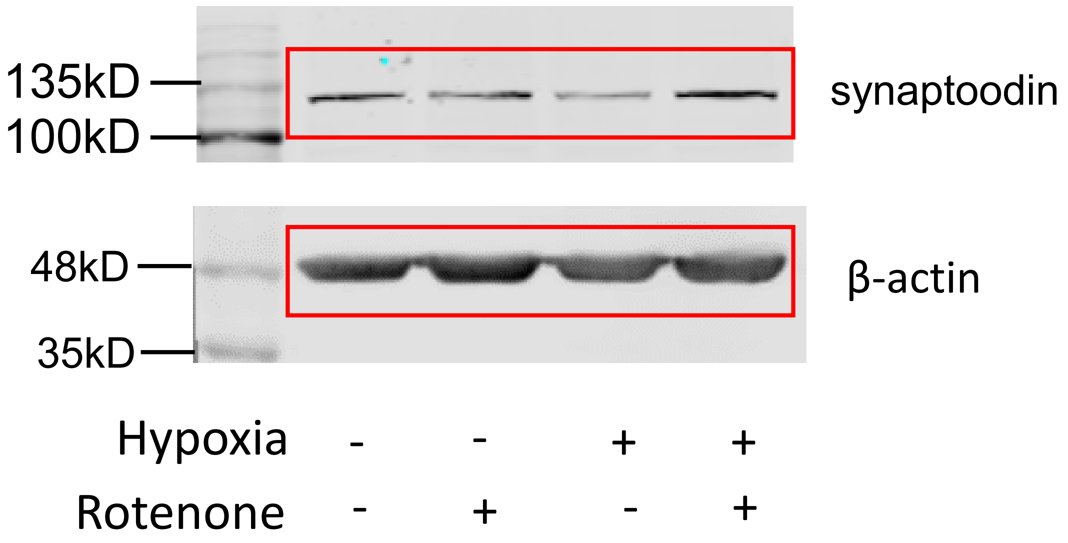

Fig. 7

A

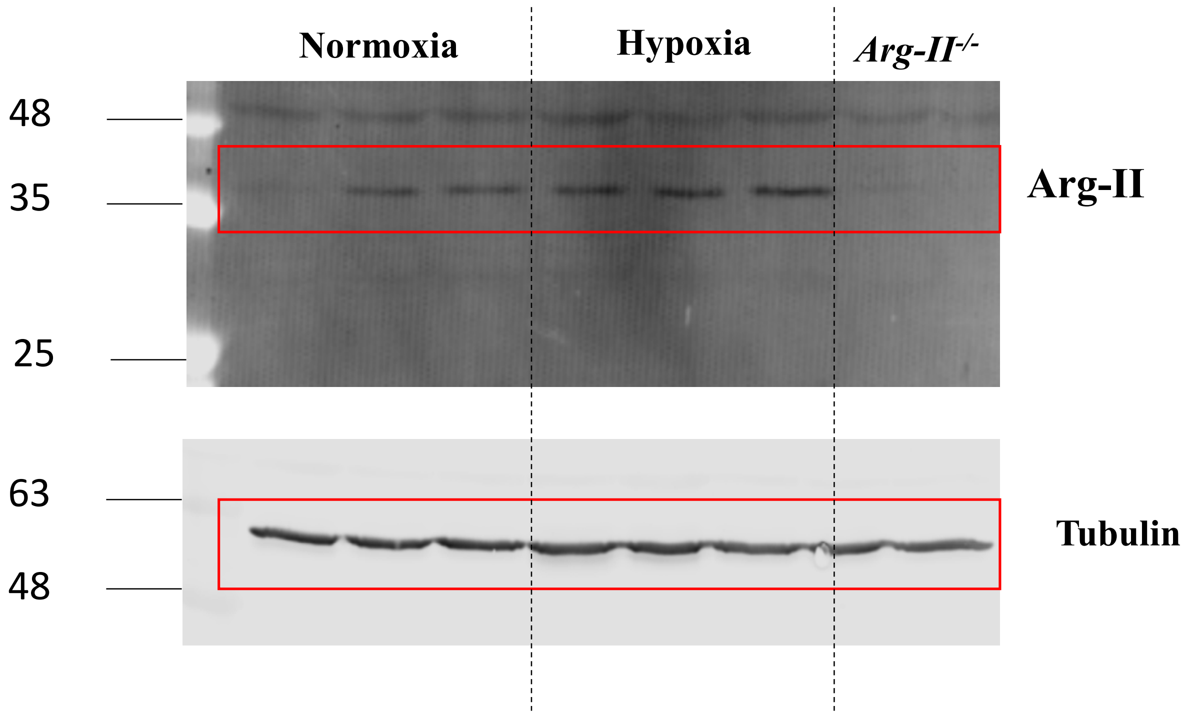

Original blot for Suppl. Fig. 1

A

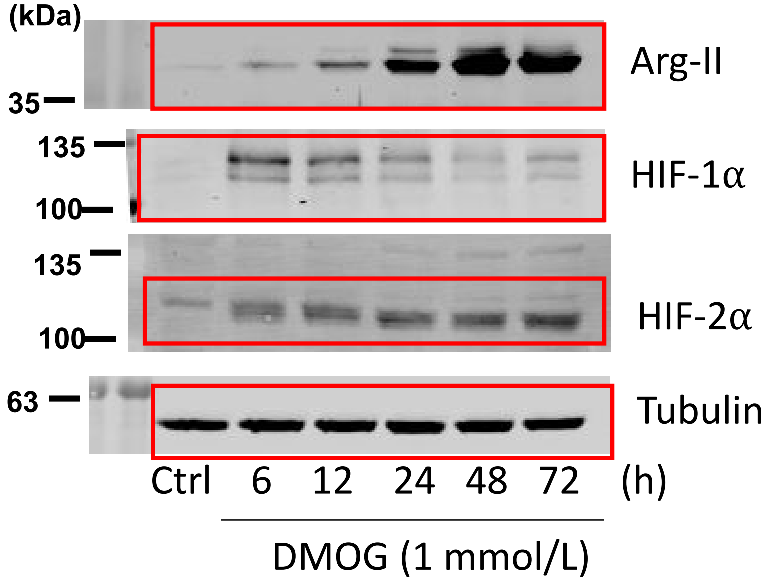

Original blot for Suppl. Fig. 5

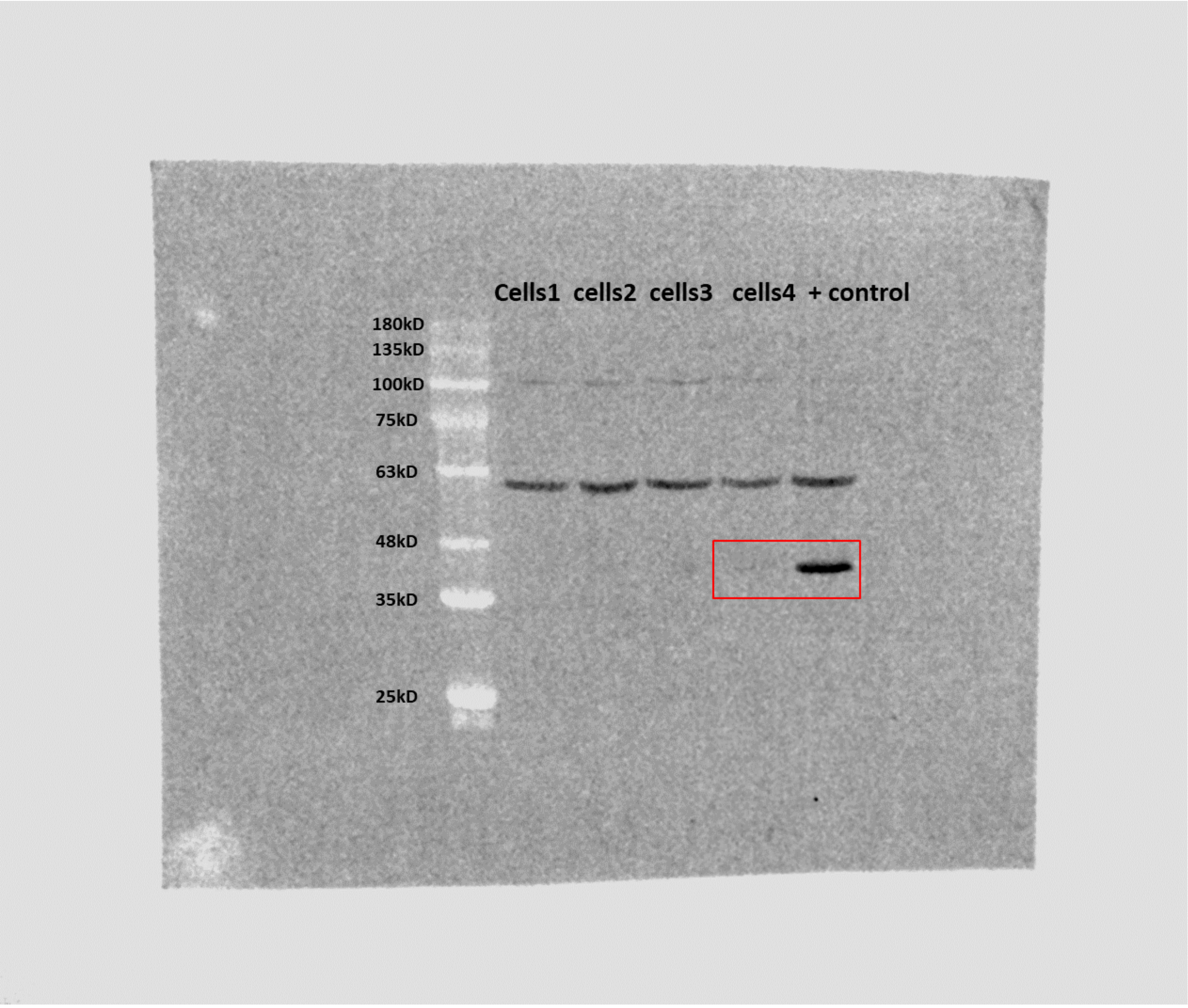

Supplement: Supplementary file 1 [file biomolecules-12-01213-s001.zip › Original blots-Final.pdf]
