# Supplementary material for: Role of Arginase-II in Podocyte Injury under Hypoxic Conditions
Source: Biomolecules. 2022 Aug 31;12(9):1213. doi: 10.3390/biom12091213 (PMC9496188; doi:10.3390/biom12091213)
Supplement: Supplementary file 1 [file biomolecules-12-01213-s001.zip › Suppl. Fig. Legends_Final.pdf]

Suppl. figure legends

Suppl. Figure S1. Effects of DMOG on Arg-II, HIF1α, and HIF2α levels in human podocytes. A. Representative immunoblotting of Arg-II, HIF1α, and HIF2α in podocytes. Tubulin serves as the loading controls. **B, C, D.** Quantification of the expression levels of Arg-II, HIF1α, and HIF2α respectively. *p<0.05, **p<0.01, ***p<0.005, ****p<0.0001 between the indicated groups. n=3.

Suppl. Figure S2. Effects of hypoxia on cytoskeletal actin derangement in human podocytes. A. Representative images showing phalloidin staining of cytoskeleton actin fibers in podocytes exposed to normoxia and hypoxia. Nucleoli were stained with DAPI (blue). **B.** Graphics below shows quantification of podocytes with disrupted cytoskeleton. *p<0.05, ****p<0.0001 between the indicated groups. n=3.

Suppl. Figure S3. Effects and silencing arg-ii on DMOG-induced cytoskeleton actin fiber derangement in podocytes for 48 hours. A. Representative images of phalloidin staining of cytoskeleton actin fibers in podocytes. Nucleoli were stained by DAPI (blue). **B.** Quantification of podocytes with disrupted cytoskeleton. ***p<0.001 between the indicated groups. n=3.

Suppl. Figure S4. Effects of silencing arg-ii on DMOG-induced mitochondrial ROS production in human podocytes. A. MitoSOX Red reagent staining was used to analyze mitochondrial ROS production in the cells transduced either with rAd/U6-lacZshRNA as controls or rAd/U6-arg-iishRNA. **B.** Quantification of relative fluorescence fold change in different groups of podocytes. **p<0.01, ***p<0.001 between indicated groups. n=4.

Suppl. Figure S5. Lack of effects of mROS inhibition on cytoskeleton actin fiber derangement in *arg-ii*$^{-/-}$ podocytes. A. immunoblotting confirming *arg-ii* knockout cell line generated by CRISPR/Cas9. **B.** *Arg-ii*$^{-/-}$ were pre-treated with or without rotenone (2 µmol/L) for 1 hour and then incubated under normoxia or hypoxia (1% O_2) condition for 24 hours. Representative images showing no effects of hypoxia and/or rotenone on cytoskeleton actin fiber derangement. Nucleoli were stained with DAPI (blue). Bar graph below shows quantification of podocytes with disrupted cytoskeleton, which remains the same in all groups. n = 3.

Suppl. Figure S6. Arg-II expression in proximal tubular cells in wild type aged mice. Confocal immunofluorescence staining of Arg-II (green) and angiotensin-converting enzyme-

1 (ACE1) (red) co-localization under hypoxic conditions. Nucleoli were stained with DAPI (blue). *wt* = wild type; The experiments were done in 3 mice.

Suppl. Figure S7. Increase in Arg-II levels in glomeruli of aged mice. Confocal immunofluorescence staining of Arg-II (green) and synaptopodin (red) co-localization with quantification of Arg-II signals. Nucleoli were stained with DAPI (blue). *wt* = wild type; n = 5, **$p<0.01$ between the indicated groups.
