# Supplementary material for: Role of Arginase-II in Podocyte Injury under Hypoxic Conditions
Source: Biomolecules. 2022 Aug 31;12(9):1213. doi: 10.3390/biom12091213 (PMC9496188; doi:10.3390/biom12091213)
Supplement: Supplementary file 1 [file biomolecules-12-01213-s001.zip › Supplemental Table S1_Final.pdf]

Supplementary Table S1. Antibody dilutions used for immunoblotting and immunofluorescence

Antibody target	Dilution
HIF1α (610958, BD Transduction Laboratories)	WB 1:1,000
HIF2α (PAB12124, Abnova)	WB 1:1’000
Arg-II (cell signaling, 55003)	WB 1:1’000;<br>IF: 1:100
ACE1 (sc-23908, Santa Cruz)	IF: 1:50
β-actin (A5441, Sigma-Aldrich)	WB 1:10’000
Tubulin (T5168, Sigma-Aldrich)	WB 1:15’000
Podocin (PA5-37284, Invitrogen / Thermo Fisher Scientific)	WB 1:1’000
Synaptopodin (sc-515842, Santa Cruz)	WB: 1:100<br>IF: 1:100
IRDye 800-conjugated affinity purified goat anti-rabbit IgG	WB 1:5,000
Alexa fluor 680-conjugated goat anti-mouse IgG	WB 1:5,000
Alexa Fluor 488-conjugated goat anti-rabbit IgG (H+L) secondary Ab	IF 1:400
Alexa Fluor 488-conjugated goat anti-mouse IgG (H+L) secondary Ab	IF 1:400
Alexa Fluor 568-conjugated goat anti-mouse IgG (H+L) secondary Ab	IF 1:400
