# Supplementary figures and images for: Role of Arginase-II in Podocyte Injury under Hypoxic Conditions
Source: Biomolecules. 2022 Aug 31;12(9):1213. doi: 10.3390/biom12091213 (PMC9496188; doi:10.3390/biom12091213)

**A**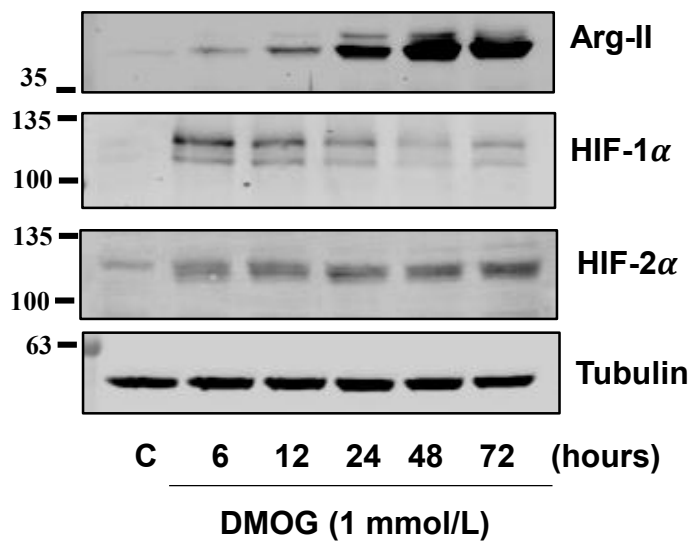**B**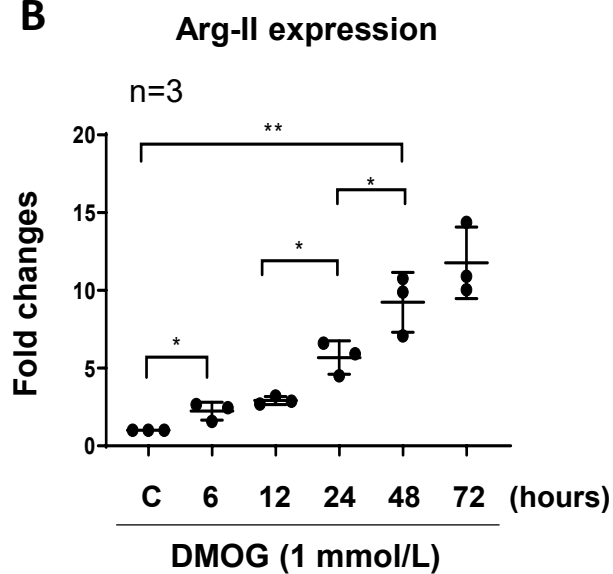**C**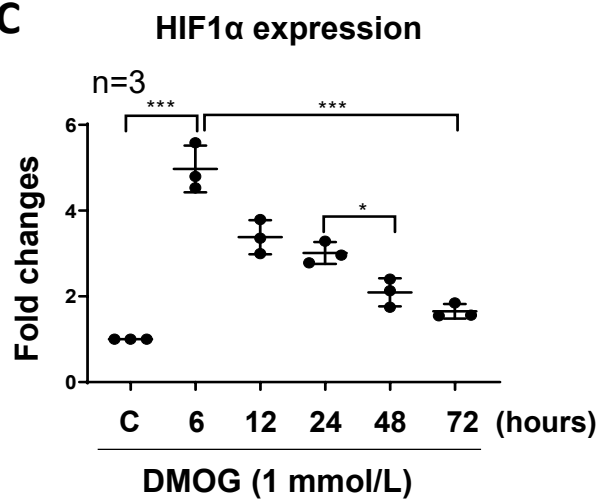**D**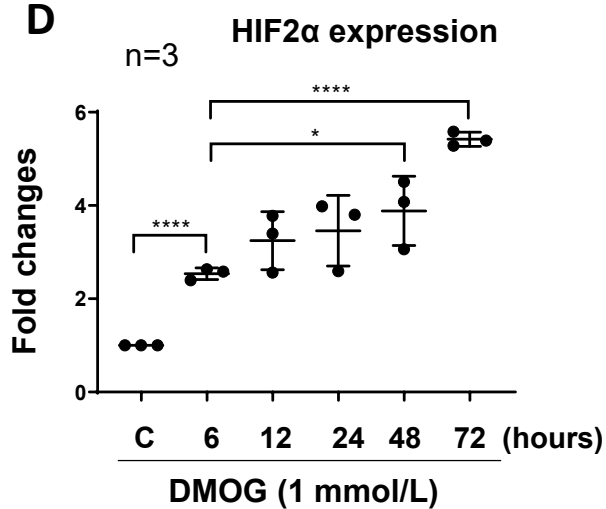

Suppl. Figure S1

Supplement: Supplementary file 1 [file biomolecules-12-01213-s001.zip › Suppl. Figure S1.pdf]

**A**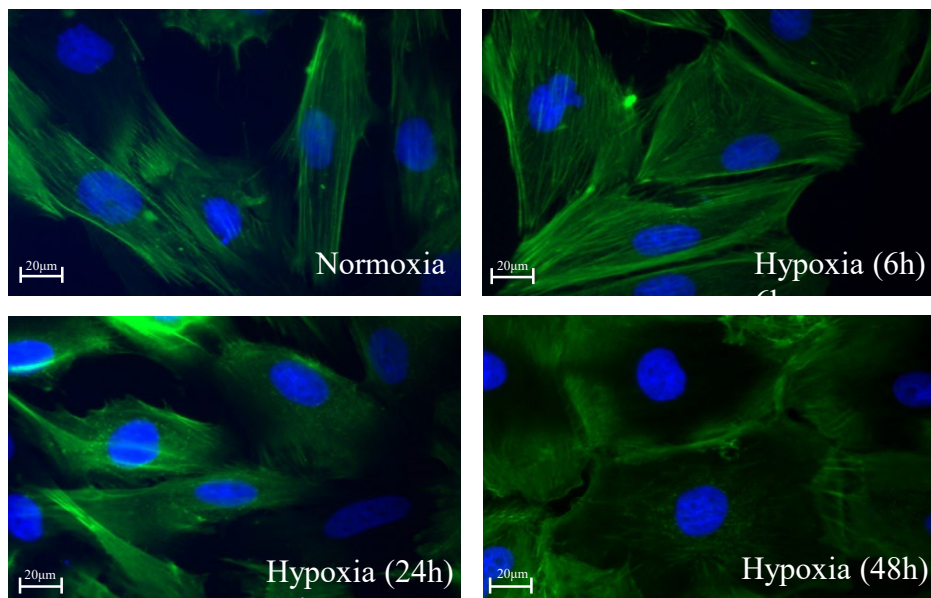**B**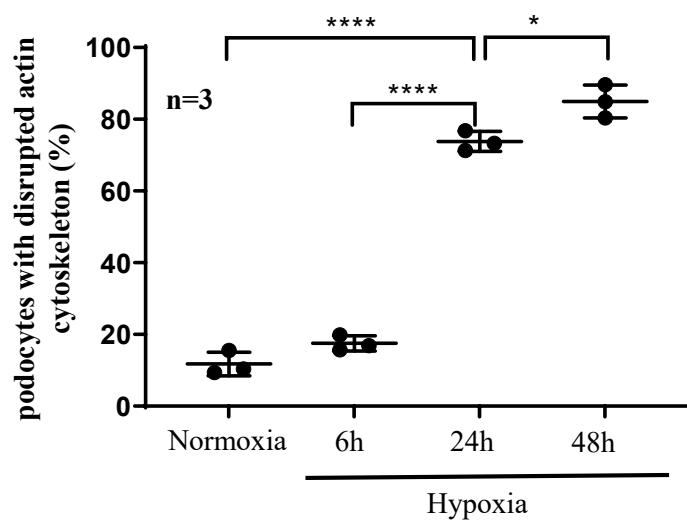

**Suppl. Figure S2**

Supplement: Supplementary file 1 [file biomolecules-12-01213-s001.zip › Suppl. Figure S2.pdf]

**A**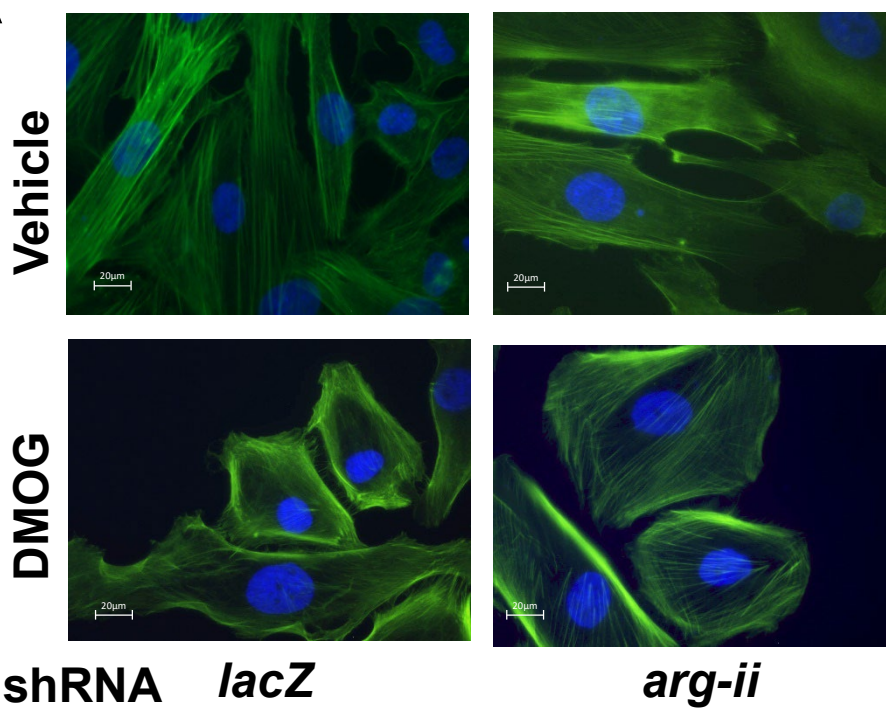**B**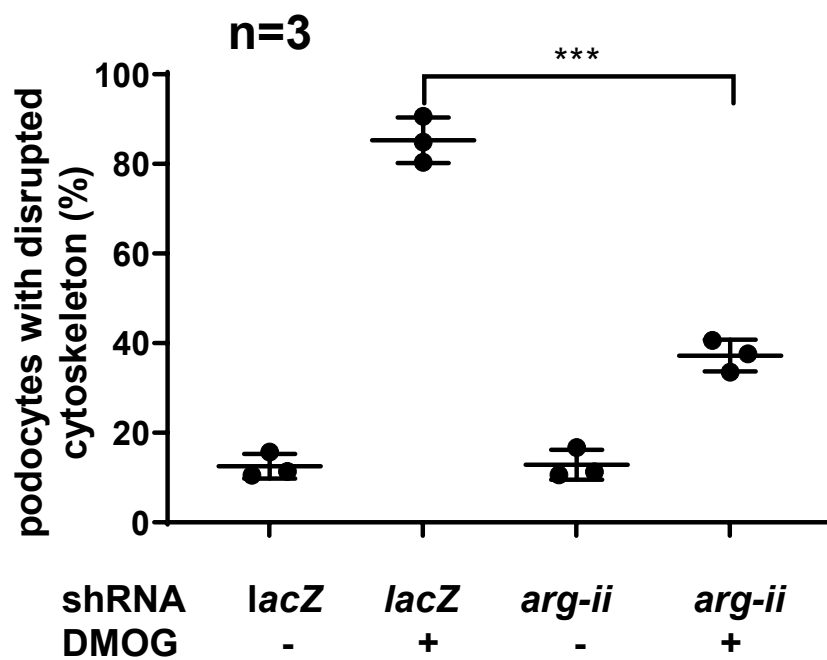

Suppl. Figure S3

Supplement: Supplementary file 1 [file biomolecules-12-01213-s001.zip › Suppl. Figure S3.pdf]

A

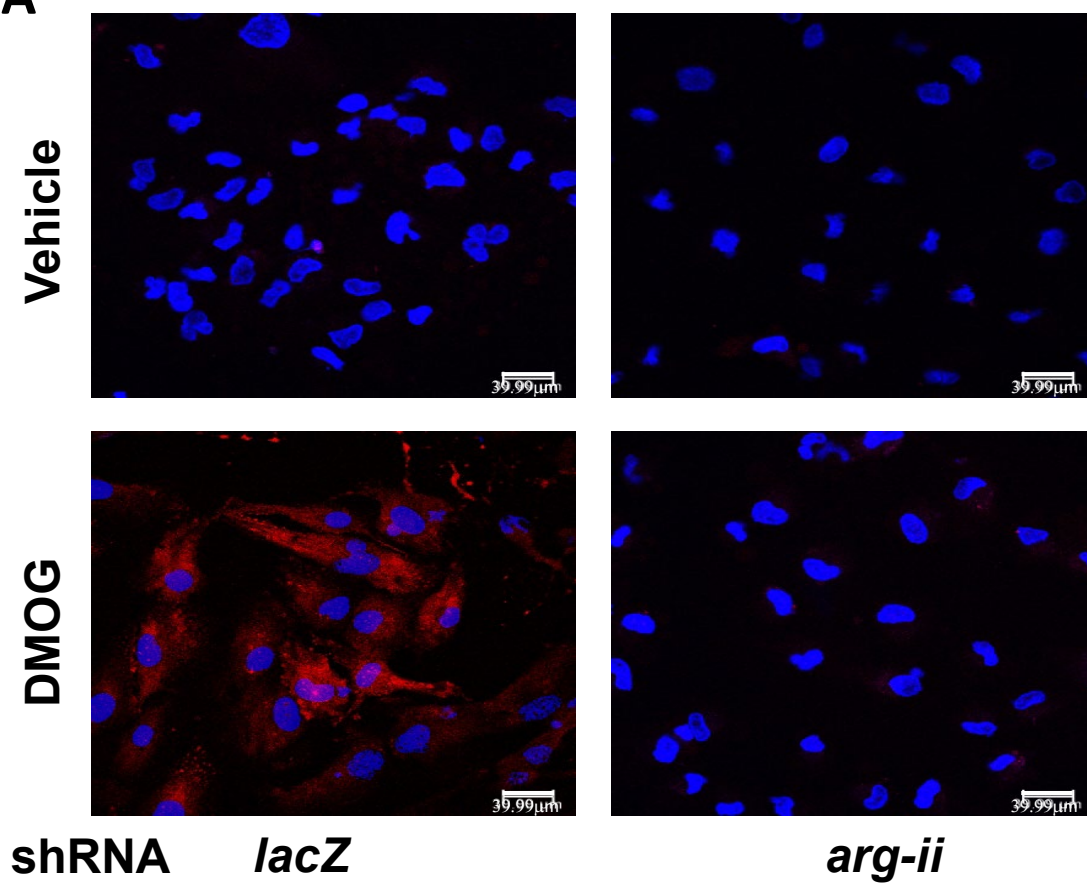

B

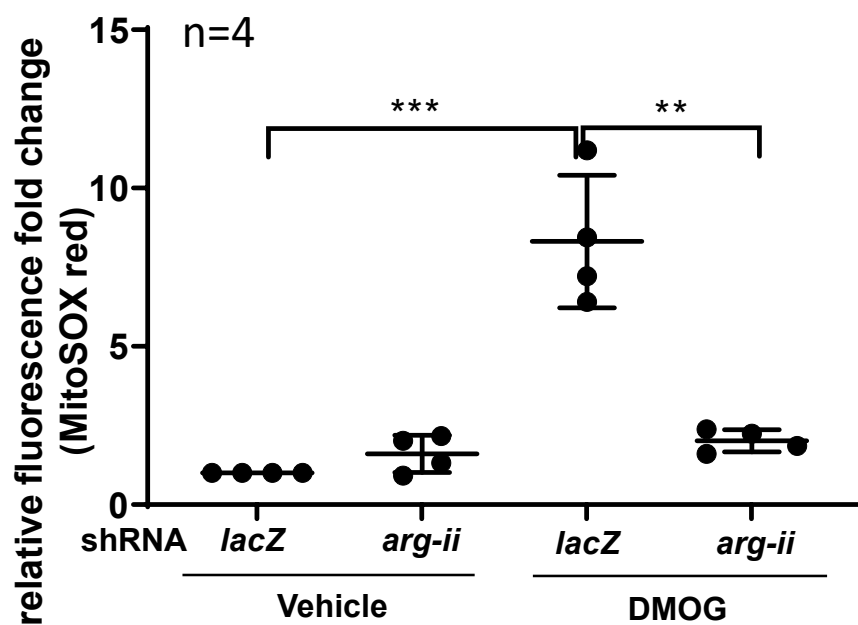

Suppl. Figure S4

Supplement: Supplementary file 1 [file biomolecules-12-01213-s001.zip › Suppl. Figure S4.pdf]

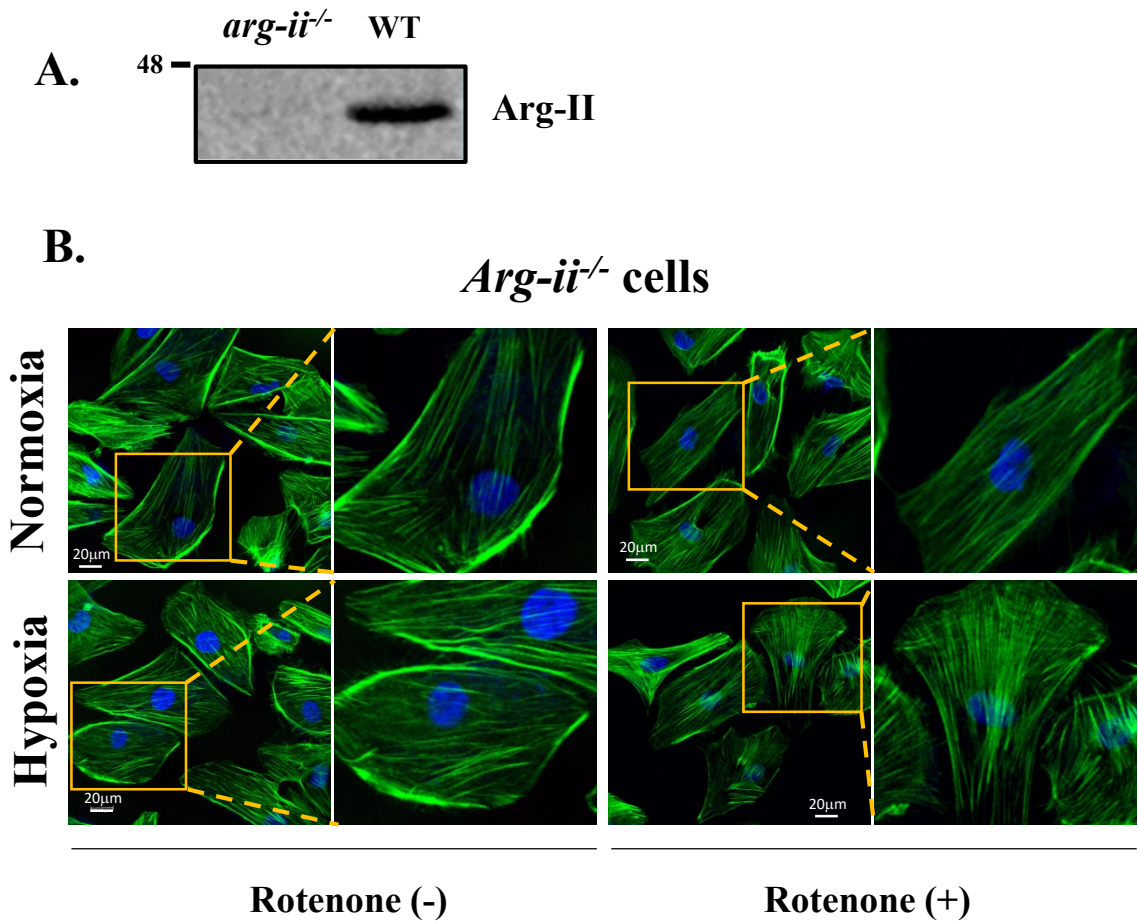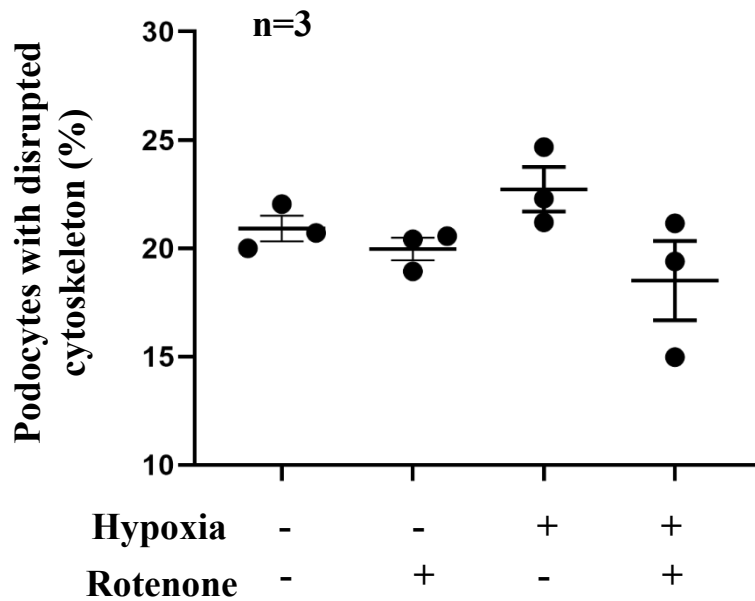

Suppl. Figure S5

Supplement: Supplementary file 1 [file biomolecules-12-01213-s001.zip › Suppl. Figure S5.pdf]

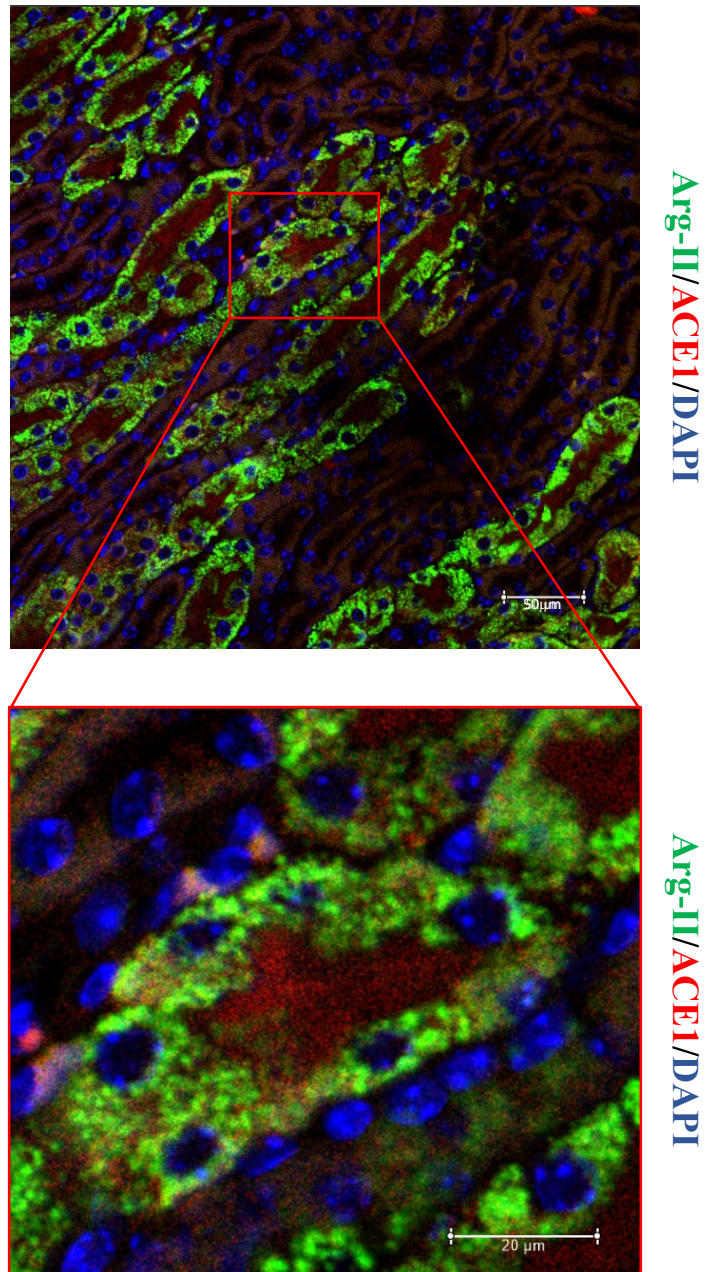

***wt* - Old (Hypoxia)**

**Suppl. Figure S6**

Supplement: Supplementary file 1 [file biomolecules-12-01213-s001.zip › Suppl. Figure S6.pdf]

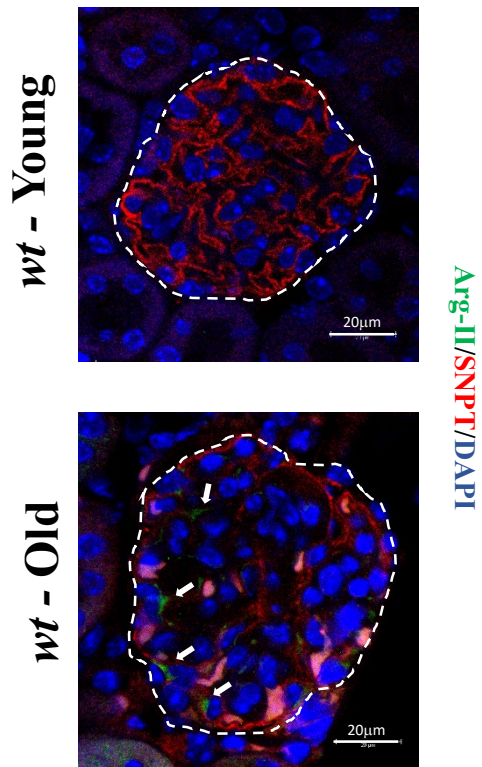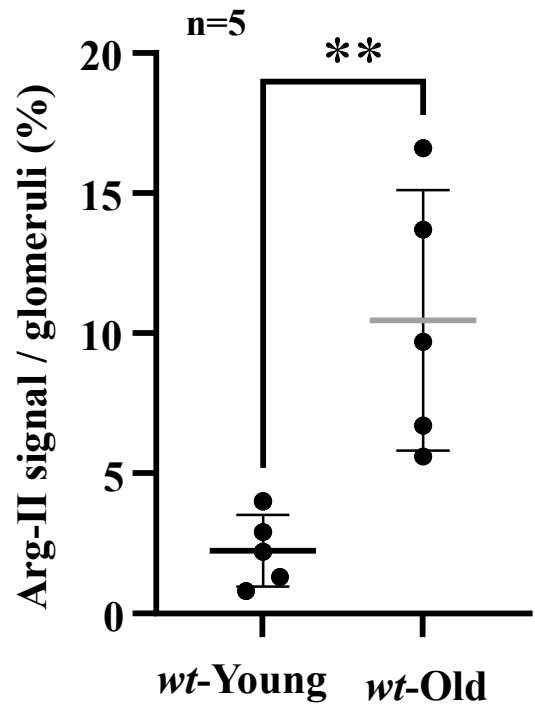

Suppl. Figure S7

Supplement: Supplementary file 1 [file biomolecules-12-01213-s001.zip › Suppl. Figure S7.pdf]
